# Supplementary material for: Investigation and research on elderly people’s willingness to combine medical and health care and related factors in coastal cities in eastern China
Source: PeerJ. 2022 Sep 7;10:e14004. doi: 10.7717/peerj.14004 (PMC9463997; doi:10.7717/peerj.14004)
Supplement: Supplemental Information 1 [file peerj-10-14004-s001.docx]

**Questionnaire of elderly people's willingness to combine medical care with health care and its influencing factors in coastal cities in eastern China**

With the deepening of the aging of population and the change of family structure, the family pension function is weakening. The way of taking care of the elderly by family can not fully adapt to the current situation of the rapid growth of the elderly population and the growing health needs of the elderly. In order to understand the experience and views of the elderly in medical treatment, pension, nursing and rehabilitation in Hangzhou, this questionnaire survey was carried out. This questionnaire is anonymous, and we will keep your answer confidential. Please feel free to fill it out. Thank you very much for your cooperation. You can put a "√" in the corresponding box.

1. **Basic information.**

| Gender: □ Male □ Female  Age：  □ 60-65 years □ 66-70 years □ 71-75 years □ 76-80 years □ >80 years  Level of education：  □ Primary school the following □ Primary school □ Junior high school □ technical secondary school □ high school □ junior college □bachelor degree or above  Household type：  □ Urban residents □ Rural farmers  Marital status：  □ Married □ Single □ Divorced □ Widowed  Children： □ A son □ A daughter □Two sons □Two daughters □A son and a daughter □childless □ others  Occupation before retirement：  □ Enterprise employees □ Civil servants □ Institutions  □ Self-employed □ Migrant workers □ Farmers □ Others  Medical treatment insurance:  □ None □ Commercial insurance □ New rural cooperative  □ Urban residents medical treatment □ Town worker medical insurance □ endowment insurance、Medical insurance  endowment Insurance:  □ None □ Agency institution □ Urban workers or urban and rural residents □ Land expropriated farmers business □ Business  Family monthly income：  □ <3000 RMB □ 3000-5999 RMB □ 6000-9999 RMB □ ≥10000 RMB |
| --- |

1. **Health status and medical care health informed of the situation**

Your physical health（ ）

1. Excellent B. Healthy C. General D.Bad E.Poor

Your living conditions（ ）

A. You can take care of yourself B. In most cases, you can do it yourself C. I can't really take care of myself D. Need someone to take care of E. Totally in need of care

Have you experienced any pain in any part of your body in the last two weeks?（ ）

A. No pain at all B. A little bit sore C. Moderate pain

D. Severe pain E. A lot of pain

Have you had enough energy to take care of your daily routine in the last two weeks?（ ）

A. Full of energy B. Better energy C. Energy is ok

D. Low energy E. There is no energy

5、For the following statements, please indicate whether you are always, often, sometimes, rarely, or never. You can draw "√" in the corresponding position

|  | Always appear | often appear | Sometimes appear | rarely | Never appear |
| --- | --- | --- | --- | --- | --- |
| Lack of security |  |  |  |  |  |
| Self sealing |  |  |  |  |  |
| Lack of living power |  |  |  |  |  |
| Difficult to cope with life changes |  |  |  |  |  |
| Big mood swings |  |  |  |  |  |

6、Have you learned about the integration of medical care, elderly care, nursing and rehabilitation（ ）

A. Know very well B. Know C. Understand better

C. Heard about it but didn't understand it D. Never heard

**三、Health intention of medical maintenance and its influencing factors**

1、Your current mode of care（ ）

A. Home care B. Community care C. Institutional care D. Mutual endowment E. Other

2、The model of care you expect（ ）（multi-select）

A. Home care B. Community care C. Institutional care D. Mutual endowment E. Other

3、You do not support the combination of medical treatment, pension, nursing and rehabilitation（ ）

A. Excellent supportive B. supportive C.General D. Don't support E. Very unsupportive

4、How much money are you willing to spend every month on medical treatment, pension, rehabilitation and nursing?（ ）（yuan/month）

A.0-999 B.1000-1999 C. 2000-2999 D.3000-3999 E.≥4000

5、Your child does not support the combination of medical treatment, pension, nursing and rehabilitation（ ）

A. Very willing to B. Willing to C. general D. Reluctant to E. Very reluctant F. childless

6、What do you think has the greatest impact on your entering a nursing home（ ）

A. children B. oneself C. spouse

D. Other family members E. professionals F. friend

7、Which facilities do you care about?（ ）（multi-select）

A. Intelligent equipment (WiFi, positioning system for the elderly, etc.)

B. Safety equipment (emergency calling system, wall handrails) C. Rehabilitation Equipment (Training ladder)

D. Medical equipment (portable oxygen tanks, first aid kit) E. Cultural and entertainment facilities (activity room, chess and card room) F. Cleaning equipment (washing machine, washing articles) G. Nutritional catering facilities (catering tools, communal kitchen)

H. Building Facilities (Barrier-free lift) I.Other

8. Please state whether you strongly disagree, somewhat disagree, generally, somewhat agree or strongly agree with the following statements. You can put "√" in the corresponding position.

|  | Couldn't agree more | agree | general | Partial disagree | Strongly disagree |
| --- | --- | --- | --- | --- | --- |
| The place where I live now is very neat and clean |  |  |  |  |  |
| It's like home where I live now |  |  |  |  |  |
| I feel my family's care for me is warm, do not feel dislike |  |  |  |  |  |
| I'm attached to my family's care services |  |  |  |  |  |

9. Please write down your suggestions on China's medical, pension, nursing and rehabilitation services

Thank you again for your support in spite of your busy schedule.
