# Supplementary material for: Investigation and research on elderly people’s willingness to combine medical and health care and related factors in coastal cities in eastern China
Source: PeerJ. 2022 Sep 7;10:e14004. doi: 10.7717/peerj.14004 (PMC9463997; doi:10.7717/peerj.14004)
Supplement: Supplemental Information 2 [file peerj-10-14004-s002.docx]

**中国东部沿海城市老年人对医养护康结合意愿及影响因素的问卷调查表**

随我国人口老龄化程度加深和家庭结构的变化，家庭养老功能不断弱化，以家庭来照顾老年人的方式已无法完全适应老年人口快速增长的现状及老年人日益增长的健康需求。为了解杭州市老年人医疗、养老、护理、康复方面的体验和看法，特开展本次问卷调查。本问卷实行匿名制，我们将对您的回答保密，请您放心填写，非常感谢您的配合。可在相应选项处画“√”。

**一、基本信息**

| 性 别： □ 男 □ 女  年 龄：  □ 60-65岁 □ 66-70岁 □ 71-75岁 □ 76-80岁 □ >80岁  文化程度：  □ 小学以下 □ 小学 □ 初中 □ 中专 □ 高中 □ 大专 □本科及以上  户籍类型：  □ 城镇居民 □ 郊区农民  婚姻状况：  □ 已婚 □ 未婚 □ 丧偶 □ 离异  您的子女情况： 个儿子， 个女儿。  退休前职业：  □ 企业员工 □ 国家公务员 □ 事业单位  □ 个体户 □ 务工 □ 务农 □ 其他  医疗保险情况：  □ 无 □ 商业保险 □ 新型农村合作  □ 城镇居民医疗保险 □ 城镇职工医疗保险 □ 商业保险+医疗保险  养老保险类型：  □ 无 □ 机关事业单位 □ 城镇职工或城乡居民 □ 被征地农民 □ 商业  家庭月收入情况：  □ <3000元 □ 3000-5999元 □ 6000-9999元 □ ≥10000元 |
| --- |

**二、健康状况及医养护康了解情况**

您的身体健康状况（ ）

A.很健康 B.健康 C.一般 D.差 E.很差

您的生活自理情况（ ）

A.完全可以自理 B.大部分情况下可以自理

C.不太能自理 D.需要人照顾 E.完全需要人照顾

最近两周内，您感觉身体哪里有疼痛吗？（ ）

A.完全没有疼痛 B.有一点疼痛 C.中等疼痛

D.严重疼痛 E.很严重疼痛

最近两周内，您是否有充沛的精力去照顾自己日常生活？（ ）

A.精力充沛 B.精力比较好 C.精力还可以

D.精力较差 E.毫无精力

5、对下列陈述，请说明您是总是出现，经常出现，有时出现，很少出现还是从未出现。可在相应位置处画“√”

|  | 总是出现 | 经常出现 | 有时出现 | 很少出现 | 从未出现 |
| --- | --- | --- | --- | --- | --- |
| 缺乏安全感 |  |  |  |  |  |
| 自我封闭 |  |  |  |  |  |
| 缺乏生活力 |  |  |  |  |  |
| 难以应对生活变化 |  |  |  |  |  |
| 情绪波动较大 |  |  |  |  |  |

6、您是否了解过医疗、养老、护理、康复一体化（ ）

A.非常了解 B.了解 C.比较了解

C.听说过但不了解 D.从未听

**三、医养护康意愿及影响因素**

1、您目前的照护模式（ ）

A.居家照护 B.社区照护 C.机构照护 D.互助养老 E.其他

2、您期望的照护模式（ ）（多选）

A.居家照护 B.社区照护 C.机构照护 D.互助养老 E.其他

3、您支持不支医疗、养老、护理、康复的结合（ ）

A.非常支持 B.支持 C.一般 D.不太支持意 E.非常不支持

4、您愿意每月花多少钱用于医疗、养老、康复、护理？（ ）（元/月）

A.0-999 B.1000-1999 C. 2000-2999 D.3000-3999 E.≥4000

5、您的子女支持不支持医疗、养老、护理、康复的结合（ ）

A.非常愿意 B.愿意 C.一般 D.不太愿意 E.非常不愿意F.无子女

6、您认为对您进入养老院影响最大的是（ ）

A.子女 B.自己 C.配偶

D.其他家庭成员 E.专业人士 F.朋友

7、您比较关注养老机构中哪些设施？（ ）（多选）

A.智能化设备（WiFi、老年人定位系统等）

B.安全设备（紧急呼叫系统、沿墙扶手） C.康复设备（训练阶梯）

D.医疗设备（便携式氧气瓶、急救箱） E.文化娱乐设施（活动室、棋牌室）

F.清洁设备（洗衣机、洗涤用品） G.营养餐饮设施（餐饮工具、公用厨房）

H.建筑设施（无障碍电梯） I.其他

8、对下列陈述，请说明您是非常不同意，比较不同意，一般，比较同意还是非常同意。可在相应位置处画“√”

|  | 非常同意 | 比较同意 | 一般 | 比较不同意 | 非常不同意 |
| --- | --- | --- | --- | --- | --- |
| 我现在住的地方是非常整洁干净的 |  |  |  |  |  |
| 我现在住的地方像家一样 |  |  |  |  |  |
| 我感到家人对我的照顾是温暖的，没有感受到嫌弃 |  |  |  |  |  |
| 我对家里人的照护服务还是比较依恋的 |  |  |  |  |  |

9、请您写下您对我国医疗、养老、护理、康复服务的建议

再次感谢您在百忙之中给予我们的支持，您辛苦了！
