# Supplementary material for: Investigation and research on elderly people’s willingness to combine medical and health care and related factors in coastal cities in eastern China
Source: PeerJ. 2022 Sep 7;10:e14004. doi: 10.7717/peerj.14004 (PMC9463997; doi:10.7717/peerj.14004)
Supplement: Supplemental Information 5 [file peerj-10-14004-s005.docx]

Residents' cognition of the combination of medical care and health care

Residents' cognition of the combination of medical care and health care is relatively high. 63.4% knew better, knew better and knew very well, 35.6% heard of it but didn't know better and never heard of it. The cognition level of residents aged 60-80 years was more than 60%, and the cognition level of residents over 80 years old was lower than that of other age groups（
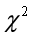
=43.925，P<0.05）；The higher the level of education, the higher the recognition of the combination of medical care and health care（
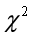
=65.490，P<0.05）；Among household registration types, urban residents have higher recognition of medical, maintenance and health combination than transfer residents（
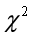
=28.331，P<0.05）；The overall cognition of married residents was significantly higher than that of other residents（
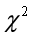
=32.027，P<0.05）；Residents whose occupations before retirement were enterprise employees and national civil servants had higher cognition levels（
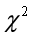
=51.861，P<0.05）；Residents with commercial insurance or commercial insurance + medical insurance had higher overall cognition than those with other medical insurance（
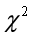
=64.252，P<0.05）；Residents without pension insurance have higher awareness than other residents（
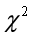
=27.857，P<0.05）；The higher the monthly household income, the higher the cognition level（
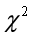
=43.312，P<0.05）。

| Understanding of the combination mode of medical care and health care | | | | | | | | |
| --- | --- | --- | --- | --- | --- | --- | --- | --- |
| Item | Number | well Known | know | better Known | Little known | unknown | 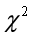 | P value |
|  |  |  |  |  |  |  |  |  |
| Gender |  |  |  |  |  |  | 8.836 | 0.087 |
| Male | 167 | 16（9.6%） | 50（29.9%） | 51（30.5%） | 28（16.8%） | 22（13.2%） |  |  |
| Female | 180 | 20（11.1%） | 46（25.6%） | 37(20.6%) | 37(20.6%) | 40（22.2%） |  |  |
| Age (years) |  |  |  |  |  |  | 43.923 | 0.000 |
| 60-65 | 130 | 17（13.1%） | 39（30.0%） | 38（29.2%） | 25（19.2%） | 11（8.5%） |  |  |
| 66-70 | 46 | 6（13.0%） | 14（30.4%） | 16（34.8%） | 5（10.9%） | 5（10.9%） |  |  |
| 71-75 | 54 | 3（5.6%） | 14（25.9%） | 18（33.3%） | 12（22.2%） | 7（13.0%） |  |  |
| 76-80 | 33 | 5（15.2%） | 9（27.3） | 5（15.2%） | 6（18.2%） | 8（24.2%） |  |  |
| >80岁 | 84 | 5（6.0%） | 20（23.8%） | 11（13.1%） | 17（20.2%） | 31（36.9%） |  |  |
| Level of education |  |  |  |  |  |  | 65.490 | 0.000 |
| Primary school the following | 44 | 2（4.5%） | 7（15.9%） | 6（13.6%） | 9（20.5%） | 20（45.5%） |  |  |
| Primary school | 52 | 1（1.9%） | 9（17.3%） | 17（32.7%） | 9（17.3%） | 16（30.8%） |  |  |
| Junior high school | 69 | 9（13.0%） | 18（26.1%） | 12（17.4%） | 15（21.7%） | 15（21.7%） |  |  |
| technical secondary school | 16 | 0(0.0% | 6（37.5%） | 4（25.0%） | 5（31.3%） | 1（6.3%） |  |  |
| high school | 60 | 9（15.0%） | 19（31.7%） | 20（33.3%） | 10（16.7%） | 2（3.3%） |  |  |
| junior college | 97 | 14（14.4%） | 34（35.1%） | 26（26.8%） | 16（16.5%） | 7（7.2%） |  |  |
| bachelor degree or above | 9 | 1（11.1%） | 3（33.3%） | 3（33.3%） | 1（11.1%） | 1（11.1%） |  |  |
| Household type |  |  |  |  |  |  | 28.331 | 0.000 |
| Urban residents | 239 | 29（12.1%） | 76（31.8%） | 63（26.4%） | 45（18.8%） | 26（10.9%） |  |  |
| Rural farmers | 108 | 7（6.5%） | 20（18.5%） | 25（23.2%） | 20（18.5%） | 36（33.3%） |  |  |
| Marital status |  |  |  |  |  |  | 32.027 | 0.012 |
| Married | 267 | 31（11.6%） | 74（27.7%） | 71（67.7%） | 55（20.6%） | 36（13.5%） |  |  |
| Single | 7 | 3（42.9%） | 0（0.0%） | 2（28.6%） | 1（14.3%） | 1（14.3%） |  |  |
| Divorced | 61 | 1（1.6%） | 18（29.5%） | 12（19.7%） | 8（13.1%） | 22（36.1%） |  |  |
| Widowed | 12 | 1（8.3%） | 4（33.3%） | 3（25.0%） | 1（8.3%） | 3（25.0%） |  |  |
| Occupation before retirement |  |  |  |  |  |  | 51.861 | 0.000 |
| Enterprise employees | 91 | 14（15.4%） | 29（31.9%） | 24（26.4%） | 19（20.9%） | 5（5.5%） |  |  |
| Civil servants | 32 | 8（25.0%） | 12（37.5%） | 4（12.5%） | 4（12.5%） | 4（12.5%） |  |  |
| Institutions | 92 | 8（8.7%） | 28（30.4%） | 27（29.3%） | 14（15.2%） | 15（16.3%） |  |  |
| Self-employed | 37 | 3（8.1%） | 11（29.7%） | 10（27.0%） | 6（16.2%） | 7（18.9%） |  |  |
| Farmers | 36 | 2（5.6%） | 9（25.0%） | 9（25.0%） | 8（22.2%） | 8（22.2%） |  |  |
| Migrant workers | 54 | 1（1.9%） | 7（13.0%） | 13（24.1%） | 12（22.2%） | 21（38.9%） |  |  |
| Others | 5 | 0（0.0%） | 0（0.0%） | 1（20.0%） | 2（40.0%） | 2（40.0%） |  |  |
| Medical treatment insurance |  |  |  |  |  |  | 64.252 | 0.000 |
| None | 23 | 2（8.7%） | 3（13.0%） | 2（8.7%） | 8（34.8%） | 8（34.8%） |  |  |
| Commercial insurance | 34 | 5（14.7%） | 16（47.1%） | 9（26.5%） | 1（2.9%） | 3（8.8%） |  |  |
| New rural cooperative | 73 | 6（8.2%） | 12（16.4%） | 16（21.9%） | 11（15.1%） | 28（38.4%） |  |  |
| urban residents medical treatment | 49 | 8（16.3%） | 10（20.4%） | 16（32.7%） | 24（28.6%） | 1（2.0%） |  |  |
| town worker medical insurance | 148 | 14（9.5%） | 49（33.1%） | 37（25.0%） | 27（18.2%） | 21（14.2%） |  |  |
| endowment insurance、Medical insurance | 20 | 1（5.0%） | 6（30.0%） | 8（40.0%） | 4（20.0%） | 1（5.0%） |  |  |
| endowment Insurance |  |  |  |  |  |  | 27.857 | 0.020 |
| None | 53 | 3（5.7%） | 18（34.0%） | 18（34.0%） | 5（9.4%） | 9（17.0%） |  |  |
| agency institution | 117 | 17（14.5%） | 33（28.2%） | 30（25.6%） | 23（19.7%） | 14（12.0%） |  |  |
| Urban workers or urban and rural residents | 125 | 14（11.2%） | 29（23.2%） | 32（25.6%） | 27（21.6%） | 23（18.4%） |  |  |
| land expropriated farmers | 26 | 1（3.8%） | 4（15.4%） | 5（19.2%） | 6（23.1%） | 10（38.5%） |  |  |
| business | 26 | 1（3.8%） | 12（46.2%） | 3（11.5%） | 4（15.4%） | 6（23.1%） |  |  |
| family monthly income |  |  |  |  |  |  |  |  |
| <3000 RMB | 46 | 3（6.5%） | 6（13.1%） | 3（6.5%） | 14（30.4%） | 20（43.5%） | 43.312 | 0.000 |
| 3000-5999 RMB | 87 | 7（8.0%） | 25（28.7%） | 23（26.4%） | 14（16.1%） | 18（20.7%） |  |  |
| 6000-9999 RMB | 110 | 12（10.9%） | 30（27.3%） | 37（33.6%） | 18（16.4%） | 13（11.8%） |  |  |
| ≥10000 RMB | 104 | 14（13.5%） | 35（33.7%） | 25（24.0%） | 19（18.3%） | 11（10.6%） |  |  |
